# Colchicine use was associated with reduced all-cause mortality in patients with diabetic gout without prior coronary artery disease: A propensity-matched nationwide cohort study

**DOI:** 10.1371/journal.pone.0362218

**Published:** 2026-11-11

**Authors:** Yonghoon Shin, Jung-Joon Cha, Ju Hyeon Kim, Hyung Joon Joo, Jae Hyoung Park, Cheol Woong Yu, Minae Park, Yujin Lee, Soon Jun Hong

**Affiliations:** 1 Department of Cardiology, Cardiovascular Center, Korea University Anam Hospital, Korea University College of Medicine, Seoul, South Korea; 2 Department of Cardiology, Asan Medical Center, University of Ulsan College of Medicine, Seoul, South Korea; 3 Department of Data Science, Hanmi Pharm. Co., Ltd, Seoul, South Korea; Fukuoka University, JAPAN

## Abstract

Colchicine has been associated with reduced cardiovascular events in secondary prevention, but evidence for primary prevention is limited. We examined the association between colchicine use and cardiovascular outcomes in patients with type 2 diabetes mellitus and gout without prior coronary artery disease. From the Korean National Health Insurance Service database, we identified 48,295 patients with diabetes and gout without prior coronary artery disease between 2015 and 2019. We compared 28,964 colchicine users with 19,331 non-users by 1:1 propensity score matching, which produced 17,337 matched pairs. The primary outcome was a composite of all-cause death, nonfatal myocardial infarction, and nonfatal stroke. The primary composite outcome occurred in 12.52% of colchicine users versus 15.03% of non-users (hazard ratio [HR] 0.71; 95% confidence interval [CI] 0.66–0.75; P < 0.001). All-cause mortality was significantly reduced in colchicine users (8.01% vs. 11.07%; HR 0.58; 95% CI 0.53–0.62; P < 0.001). No significant differences were observed for myocardial infarction (HR 1.15; 95% CI 0.96–1.37; P = 0.121) or stroke (HR 0.91; 95% CI 0.81–1.01; P = 0.076). In a landmark analysis at 365 days that addressed immortal time bias, the associations with all-cause mortality (HR 0.67; 95% CI 0.62–0.73; P < 0.001) and with the composite outcome (HR 0.76; 95% CI 0.71–0.81; P < 0.001) remained significant, whereas stroke (HR 0.99; 95% CI 0.87–1.12; P = 0.823) and myocardial infarction (HR 1.15; 95% CI 0.95–1.40; P = 0.144) did not differ between the two groups. In patients with diabetes and gout without prior coronary artery disease, colchicine use was associated with lower all-cause mortality. The reduction in the composite outcome was driven by mortality rather than by myocardial infarction or stroke.

## Introduction

Colchicine is a potent anti-inflammatory agent [1–4]. Previous studies have demonstrated that this drug has efficacy in secondary prevention of major adverse cardiovascular events (MACE) following myocardial infarction [5–7]. Based on this evidence, the 2024 European Society of Cardiology guidelines upgraded colchicine to a Class IIa recommendation for secondary cardiovascular prevention in patients with chronic coronary syndromes [8]. However, evidence for colchicine in primary prevention in patients without atherosclerotic cardiovascular disease (ASCVD) is limited [9]. Colchicine is widely used for gout management, both for acute flare treatment and prophylaxis during urate-lowering therapy initiation [10–12]. Both gout and type 2 diabetes mellitus independently increase cardiovascular risk [13–19]. We conducted a nationwide, population-based cohort study using the Korean National Health Insurance Service (NHIS) database to analyze the association between colchicine use and risk of MACE and all-cause mortality in patients with both type 2 diabetes mellitus and gout without prior coronary artery disease.

## Methods

### Study design and data source

We conducted a nationwide, population-based retrospective cohort study using the Korean NHIS database. The NHIS provides mandatory universal health insurance coverage for approximately 52 million South Korean residents, capturing comprehensive data on all healthcare utilization including diagnoses, procedures, and prescriptions. This study was approved by the Institutional Review Board of Korea University Hospital (approval number: 2024AN0026). The requirement for informed consent was waived due to the retrospective nature of the study and the use of de-identified administrative data. All study procedures were conducted in accordance with the ethical standards of the institutional research committee and the 1964 Helsinki Declaration and its later amendments.

### Study population

From the Korean NHIS database, which covers the entire Korean population and has been validated for cardiovascular epidemiological research, we identified 1,448,321 patients aged 20 years or older who were newly diagnosed with type 2 diabetes mellitus using the International Classification of Disease, 10th Revision (ICD-10) codes E11-E12 and received antidiabetic medication prescriptions between January 1, 2015, and December 31, 2019 (Fig 1) [20–23]. Among these diabetic patients, 230,331 were diagnosed with gout, defined by ICD-10 code M10 with at least two outpatient visits or one hospitalization during the study period. The index date was defined as the date of gout diagnosis.

**Fig 1:**
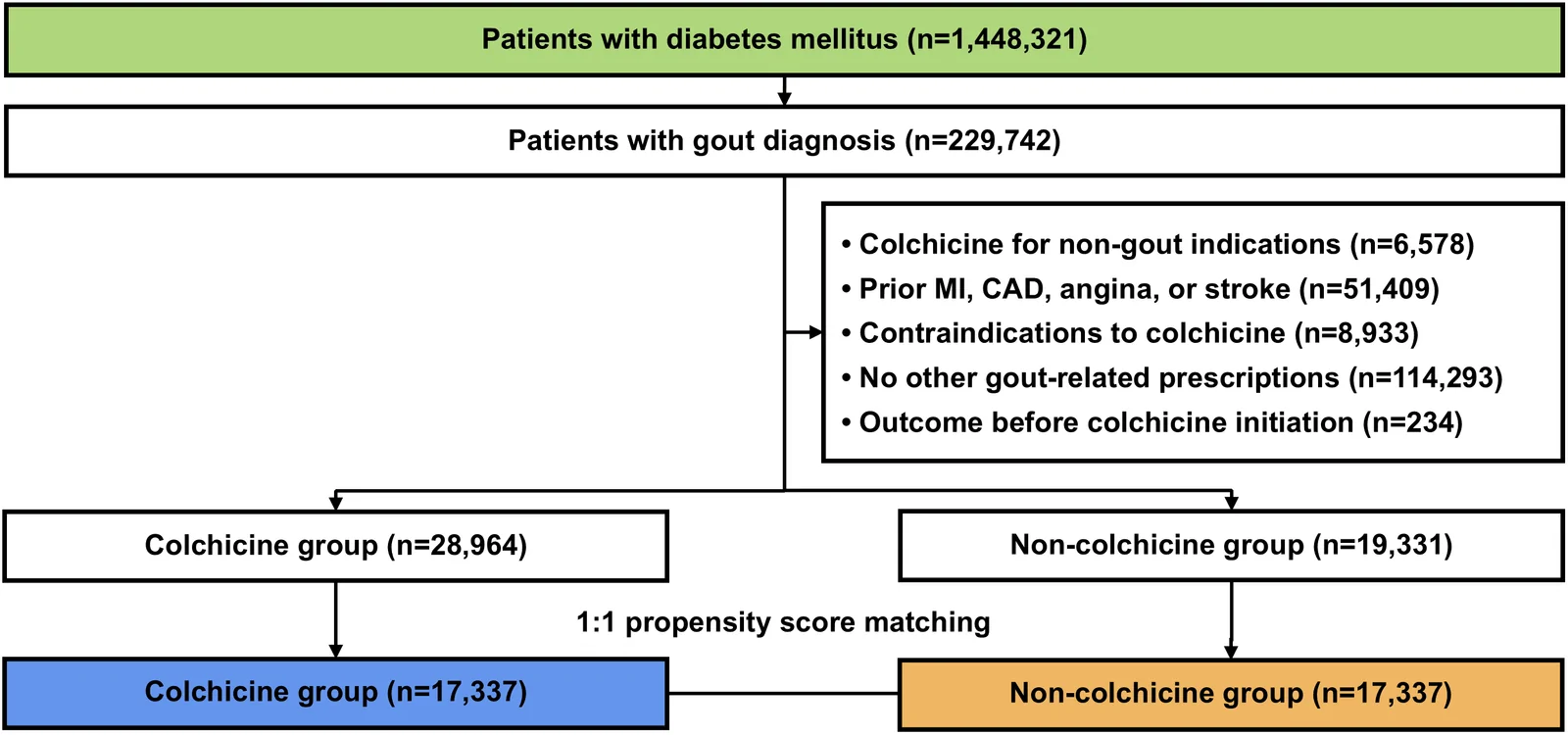
Study flowchart.

We excluded patients who met the following criteria. First, we excluded patients who had colchicine prescriptions for indications other than gout between 2013 and 2023, including pericarditis, pericardial effusion, calcium pyrophosphate deposition disease, systemic sclerosis, Behcet's disease, or primary biliary cirrhosis, identified by ICD-10 codes I30, I31, I32, I01, I09, M11, M34, M352, C7988, and K7439, resulting in exclusion of 6,578 patients. Second, we excluded 51,409 patients with prior coronary artery disease before gout diagnosis, identified by ICD-10 codes I20 through I25, which cover myocardial infarction, angina, and other ischemic heart diseases. Patients with prior stroke, other cerebrovascular disease, or peripheral artery disease were not excluded. Third, patients with absolute contraindications to colchicine use were excluded, including those with pregnancy, liver cirrhosis, or end-stage renal disease, identified by ICD-10 codes starting with O, K74, K703, N185, and Z348, excluding 8,933 patients. Fourth, we excluded 33 patients with data inconsistencies. Fifth, 556 patients who received colchicine prescriptions before their gout diagnosis were excluded. Sixth, we excluded 234 patients who experienced outcome events between their gout diagnosis and colchicine initiation. Patients with rheumatic fever (I01) and rheumatic heart disease (I09) were excluded because colchicine may be prescribed for rheumatic pericarditis in these conditions, which would confound the assessment of colchicine use for gout. Finally, we excluded 114,293 patients who received a gout diagnosis but had no prescriptions for any gout-related medications, including colchicine, febuxostat, allopurinol, or probenecid, during the study period. After these exclusions, 48,295 patients remained in the study cohort.

Among the 48,295 patients in the study cohort, 28,964 patients received at least one colchicine prescription after gout diagnosis, while 19,331 patients had no colchicine prescriptions during the follow-up period. To minimize confounding and ensure comparability between groups, we performed 1:1 propensity score matching (PSM) between colchicine users and non-users, resulting in 17,337 matched pairs for the primary analysis.

### Outcomes

The primary outcome was a composite of MACE, including all-cause death, nonfatal myocardial infarction, and nonfatal stroke. Secondary outcomes were the individual components of MACE, including all-cause death, nonfatal myocardial infarction, and nonfatal stroke. Nonfatal myocardial infarction and nonfatal stroke were identified using ICD-10 codes from hospitalization records for myocardial infarction (I21) and stroke (I63, I64). All-cause mortality was identified from the National Death Registry.

### Statistical analysis

All baseline variables used in the propensity score model were derived from insurance eligibility records and administrative claims, which provide complete information for all enrolled beneficiaries without missing data. Baseline characteristics were compared between colchicine users and non-users using standardized mean differences (SMD), with SMD < 0.1 indicating adequate balance. To reduce confounding, we performed 1:1 propensity score matching (PSM) using the nearest neighbor method with a caliper width of 0.2 standard deviations of the logit of the propensity score.

The propensity score was estimated using a logistic regression model that included age, sex, income level, region of residence, baseline comorbidities (hypertension, dyslipidemia, chronic kidney disease, heart failure, peripheral artery disease, atrial fibrillation), and baseline medications (statins, antiplatelet agents, renin-angiotensin system inhibitors, beta-blockers, metformin, sulfonylureas, insulin, urate-lowering therapy).

In the matched cohort, we estimated the risk of outcomes using Kaplan-Meier survival curves and Cox proportional hazards models. Follow-up for both groups began at the gout diagnosis date and continued until the occurrence of the outcome event, death, loss of insurance eligibility, or December 31, 2023, whichever occurred first. Hazard ratios (HR) and 95% confidence intervals (CI) were calculated, with adjustment for residual confounding if necessary. To assess how sensitive our findings were to potential unmeasured confounding, we calculated E-values for the primary composite outcome and all-cause mortality. Additionally, to address residual covariate imbalance after PSM (standardized mean difference > 0.1 for age), we performed a doubly robust analysis by further adjusting for age and sex in a multivariable Cox proportional hazards model within the matched cohort.

To address the possibility of immortal time bias, we performed a landmark analysis with a landmark time of 365 days after the gout diagnosis. Patients who died or developed the outcome of interest before the landmark were excluded from both groups. Because the number of patients at risk differed by outcome, we constructed a separate landmark cohort for each outcome and repeated 1:1 propensity score matching within each cohort with the same covariates as the primary analysis. Follow-up started at the landmark and continued until the outcome event, death, loss of insurance eligibility, or December 31, 2023, whichever occurred first. Deaths after the landmark were censored in the analyses of stroke and myocardial infarction.

All statistical analyses were performed using SAS version 9.4 (SAS Institute Inc., Cary, NC, USA). Two-sided P values <0.05 were considered statistically significant.

## Results

### Baseline characteristics

Before propensity score matching, several differences existed between colchicine users and non-users (Table 1). Colchicine users were younger (mean age 55.90 ± 11.52 years vs. 59.46 ± 11.80 years, P < 0.001) and more likely to be male (91.89% vs. 77.33%, P < 0.001). Colchicine users had lower prevalence of hypertension (52.5% vs. 64.93%, P < 0.001), dyslipidemia (30.64% vs. 44.19%, P < 0.001), chronic kidney disease (2.62% vs. 6.77%, P < 0.001), and cancer (3.69% vs. 8.46%, P < 0.001). Laboratory values showed that colchicine users had higher total cholesterol (208.16 ± 48.21 mg/dL vs. 200.30 ± 44.96 mg/dL, P < 0.001), triglycerides (247.42 ± 200.28 mg/dL vs. 212.75 ± 166.30 mg/dL, P < 0.001), and body weight (76.53 ± 13.11 kg vs. 73.62 ± 13.52 kg, P < 0.001).

**Table 1:** Baseline characteristics.

		Before Matching					After Matching				
		Colchicine user (N=28,964)	Non-user (N=19,331)	*P* value	*Missing, n*	*SMD*	Colchicine user (N=17,337)	Non-user (N=17,337)	*P* value	*Missing, n*	*SMD*
**Demographic data**											
**Age, year**		**55.90 ± 11.52**	**59.46 ± 11.80**	**<.0001**		**−0.30**	**56.72 ± 11.88**	**58.63 ± 11.60**	**<.0001**		**−0.16**
**Male sex*, n (%)***		**26,616 (91.89)**	**14949 (77.33)**	**<.0001**		**0.41**	**15,000 (86.52)**	**14,781 (85.26)**	**0.0007**		**0.04**
**Height, cm**		**168.23 ± 7.59**	**165.59 ± 8.84**	**<.0001**	**15,444**	**0.32**	**167.47 ± 8.20**	**166.84 ± 8.19**	**<.0001**	**10,733**	**0.08**
**Weight, kg**		**76.53 ± 13.11**	**73.62 ± 13.52**	**<.0001**	**15,444**	**0.22**	**76.01 ± 13.46**	**74.74 ± 13.31**	**<.0001**	**10,733**	**0.09**
**Waist circumference, cm**		**90.81 ± 10.83**	**89.83 ± 9.14**	**<.0001**	**15,447**	**0.10**	**90.85 ± 12.15**	**90.20 ± 9.01**	**<.0001**	**10,735**	**0.06**
**Systolic blood pressure, mmHg**		**130.88 ± 15.10**	**130.29 ± 15.56**	**0.0006**	**15,471**	**0.04**	**130.99 ± 15.07**	**130.28 ± 15.56**	**0.0004**	**10,755**	**0.05**
**Diastolic blood pressure, mmHg**		**81.75 ± 10.65**	**80.40 ± 10.75**	**<.0001**	**15,471**	**0.13**	**81.58 ± 10.71**	**80.67 ± 10.78**	**<.0001**	**10,755**	**0.09**
**Current smoker*, n (%)***		**7,119 (36.91)**	**3,935 (29.06)**	**<.0001**	**15,465**	**0.17**	**4,105 (34.76)**	**3,851 (31.79)**	**<.0001**	**10,752**	**0.06**
**Current alcohol use*, n (%)***		**12,937 (67.06)**	**7,497 (55.28)**	**<.0001**	**15,441**	**0.24**	**7,570 (64.08)**	**7,259 (59.84)**	**<.0001**	**10,730**	**0.09**
**Regular exercise*, n (%)***		**10,637 (55.13)**	**7,061 (52.06)**	**<.0001**	**15,439**	**0.06**	**6,370 (53.92)**	**6,484 (53.45)**	**0.4623**	**10,730**	**0.01**
**Income**	**Very low (0−4)**	**5,363 (18.80)**	**4,054 (21.28)**	**<.0001**	**712**		**3,314 (19.40)**	**3,533 (20.66)**	**0.0037**	**493**	
	**Low (5−9)**	**5,151 (18.05)**	**3,246 (17.04)**	.			**2,984 (17.47)**	**2,943 (17.21)**	.		
	**High (10−14)**	**6,820 (23.90)**	**4,214 (22.12)**	.			**4,048 (23.70)**	**3,831 (22.41)**	.		
	**Very high (15−20)**	**11,196 (39.24)**	**7,539 (39.57)**	.			**6,737 (39.44)**	**6,791 (39.72)**	.		
**CCI**	**0**	**28,292 (97.68)**	**17,842 (92.30)**	**<.0001**		**−0.22**	**16,815 (96.99)**	**16,205 (93.47)**	**<.0001**		**-0.14**
	**1**	**303 (1.05)**	**373 (1.93)**	.			**208 (1.20)**	**306 (1.77)**	.		
	**≥2**	**369 (1.27)**	**1,116 (5.77)**	.			**314 (1.81)**	**826 (4.76)**	.		
**Past medical history, n (%)**											
**Heart failure**		**1,136 (3.92)**	**1,139 (5.89)**	**<.0001**		**0.09**	**940 (5.42)**	**893 (5.15)**	**0.2593**		**−0.01**
**Hemorrhagic Stroke**		**134 (0.46)**	**156 (0.81)**	**<.0001**		**−0.04**	**88 (0.51)**	**129 (0.74)**	**0.0052**		**−0.03**
**Ischemic Stroke**		**830 (2.87)**	**842 (4.36)**	**<.0001**		**−0.08**	**609 (3.51)**	**707 (4.08)**	**0.0059**		**−0.03**
**Hypertension**		**15,207 (52.50)**	**12,552 (64.93)**	**<.0001**		**0.25**	**10,282 (59.31)**	**10,952 (63.17)**	**<.0001**		**0.08**
**Dyslipidemia**		**8,876 (30.64)**	**8542 (44.19)**	**<.0001**		**0.28**	**6,712 (38.71)**	**7,254 (41.84)**	**<.0001**		**0.07**
**Chronic lung disease**		**6,866 (23.71)**	**5,684 (29.40)**	**<.0001**		**−0.13**	**4,433 (25.57)**	**4,799 (27.68)**	**<.0001**		**−0.05**
**Chronic liver disease**		**51 (0.18)**	**49 (0.25)**	**0.0668**		**−0.02**	**33 (0.19)**	**40 (0.23)**	**0.4121**		**−0.01**
**Chronic kidney disease**		**758 (2.62)**	**1,308 (6.77)**	**<.0001**		**−0.20**	**545 (3.14)**	**1,107 (6.39)**	**<.0001**		**−0.15**
**End staged kidney disease**		**25 (0.09)**	**90 (0.47)**	**<.0001**		**−0.07**	**17 (0.10)**	**77 (0.44)**	**<.0001**		**−0.07**
**Cerebrovascular disease**		**1,827 (6.31)**	**1,855 (9.60)**	**<.0001**		**−0.12**	**1,300 (7.50)**	**1,547 (8.92)**	**<.0001**		**−0.05**
**Peripheral artery disease**		**3,386 (11.69)**	**3,190 (16.50)**	**<.0001**		**0.14**	**2,380 (13.73)**	**2,722 (15.70)**	**<.0001**		**0.06**
**Cancer**		**1,068 (3.69)**	**1,635 (8.46)**	**<.0001**		**0.20**	**970 (5.59)**	**1,121 (6.47)**	**0.0007**		**0.04**
**Valvular heart disease**		**179 (0.62)**	**135 (0.70)**	**0.2817**		**−0.01**	**150 (0.87)**	**101 (0.58)**	**0.0019**		**0.03**
**Arrhythmia**		**930 (3.21)**	**831 (4.30)**	**<.0001**		**0.06**	**868 (5.01)**	**684 (3.95)**	**<.0001**		**−0.06**
**Atrial fibrillation**		**561 (1.94)**	**484 (2.50)**	**<.0001**		**0.04**	**521 (3.01)**	**404 (2.33)**	**<.0001**		**−0.05**
**Laboratory results, means ± standard deviations**											
**Fasting plasma glucose**		**120.48 ± 34.96**	**122.08 ± 39.43**	**0.0002**	**15,470**	**−0.04**	**119.85 ± 34.31**	**122.80 ± 39.39**	**<.0001**	**10,754**	**−0.08**
**Total cholesterol**		**208.16 ± 48.21**	**200.30 ± 44.96**	**<.0001**	**15,681**	**0.17**	**207.65 ± 51.04**	**200.32 ± 44.85**	**<.0001**	**10,923**	**0.15**
**Low-density lipoprotein**		**115.81 ± 45.87**	**111.75 ± 39.84**	**<.0001**	**17,543**	**0.09**	**115.26 ± 47.09**	**111.53 ± 39.67**	**<.0001**	**12,239**	**0.09**
**High-density lipoprotein**		**48.09 ± 12.91**	**49.11 ± 13.88**	**<.0001**	**15,681**	**−0.08**	**48.48 ± 12.63**	**48.70 ± 13.87**	**0.2098**	**10,923**	**−0.02**
**Triglycerides**		**247.42 ± 200.28**	**212.75 ± 166.30**	**<.0001**	**15,681**	**0.19**	**243.90 ± 195.88**	**217.32 ± 171.05**	**<.0001**	**10,923**	**0.14**
**Aspartate aminotransferase**		**34.79 ± 25.40**	**33.67 ± 25.39**	**<.0001**	**15,470**	**0.04**	**34.69 ± 26.36**	**34.04 ± 25.93**	**0.0546**	**10,754**	**0.02**
**Alanine aminotransferase**		**40.61 ± 34.50**	**37.58 ± 30.27**	**<.0001**	**15,470**	**0.09**	**40.19 ± 36.62**	**38.44 ± 30.61**	**<.0001**	**10,754**	**0.05**
**Gamma-glutamyl transferase**		**83.24 ± 90.14**	**70.83 ± 86.00**	**<.0001**	**15,470**	**0.14**	**81.15 ± 88.34**	**74.16 ± 88.08**	**<.0001**	**10,754**	**0.08**
**Medication**											
**DOAC**		**2,015 (6.96)**	**1,589 (8.22)**	**<.0001**		**−0.05**	**1,448 (8.35)**	**1,279 (7.38)**	**0.0007**		**0.04**
**Vitamin K antagonist**		**652 (2.25)**	**431 (2.23)**	**0.8758**		**0.00**	**499 (2.88)**	**349 (2.01)**	**<.0001**		**0.06**
**Aspirin**		**10,967 (37.86)**	**7,856 (40.64)**	**<.0001**		**−0.06**	**6,888 (39.73)**	**6,879 (39.68)**	**0.9213**		**0.00**
**Clopidogrel**		**5,601 (19.34)**	**4,006 (20.72)**	**0.0002**		**−0.03**	**3,482 (20.08)**	**3,505 (20.22)**	**0.7581**		**0.00**
**Anti-platelet**		**6,912 (23.86)**	**4,990 (25.81)**	**<.0001**		**−0.05**	**4,333 (24.99)**	**4,328 (24.96)**	**0.9505**		**0.00**
**P2Y12 inhibitor**		**608 (2.10)**	**265 (1.37)**	**<.0001**		**0.06**	**332 (1.91)**	**248 (1.43)**	**0.0004**		**0.04**
**Lipid-lowering agents**		**26,160 (90.32)**	**17,343 (89.72)**	**0.0299**		**0.02**	**15754 (90.87)**	**15,544 (89.66)**	**0.0001**		**0.04**
**Beta-blocker**		**11,910 (41.12)**	**8,764 (45.34)**	**<.0001**		**−0.09**	**7,612 (43.91)**	**7,605 (43.87)**	**0.9396**		**0.00**
**SGLT2 inhibitor**		**8,471 (29.25)**	**5,102 (26.39)**	**<.0001**		**0.06**	**4,978 (28.71)**	**4,719 (27.22)**	**0.0019**		**0.03**
**ARB**		**22,183 (76.59)**	**15,000 (77.60)**	**0.0100**		**−0.02**	**13,637 (78.66)**	**13,380 (77.18)**	**0.0009**		**0.04**
**ACE inhibitor**		**1,544 (5.33)**	**1,098 (5.68)**	**0.0982**		**−0.02**	**991 (5.72)**	**970 (5.59)**	**0.6254**		**0.01**
**Diuretics**		**9,662 (33.36)**	**6,825 (35.31)**	**<.0001**		**−0.04**	**6,201 (35.77)**	**5,952 (34.33)**	**0.0051**		**0.03**

* Data are presented as means ± standard deviations, medians (IQR), or n (%).

* Propensity score matching was performed based on age, sex, income level, region of residence, baseline comorbidities (hypertension, dyslipidemia, CKD, HF, PAD, arrhythmia, AF), baseline medications (statins, antiplatelet agents, RAS inhibitors, beta-blockers, metformin, sulfonylureas, insulin, urate-lowering therapy).

* ACE, angiotensin-converting enzyme; ARB, angiotensin receptor blocker; CCI, Charlson comorbidity index; DOAC, direct oral anticoagulant; SGLT2, sodium-glucose cotransporter-2.

After 1:1 propensity score matching based on age group, sex, heart failure history, hypertension, dyslipidemia, peripheral artery disease, cancer, arrhythmia, and atrial fibrillation, 17,337 matched pairs were included in the primary analysis. The matching procedure achieved good balance between groups, with standardized mean differences less than 0.1 for most matched variables. After matching, the mean age was 56.72 ± 11.88 years in colchicine users and 58.63 ± 11.60 years in non-users (SMD = −0.16). The proportion of men was 86.52% in colchicine users and 85.26% in non-users (SMD = 0.04). Baseline comorbidities were well balanced, including hypertension (59.31% vs. 63.17%) and dyslipidemia (38.71% vs. 41.84%). Among colchicine users, the mean total prescription duration was 192.12 days (standard deviation, 406.01 days) (Table A in S1 File).

### Outcomes

The primary composite outcome occurred in 2,170 colchicine users (12.52%) and 2,606 non-users (15.03%). Colchicine use was associated with a significant 29% reduction in the primary outcome (HR 0.71; 95% CI 0.66–0.75; P < 0.001) (Fig 2, Table 2). Among the individual components of the primary outcome, all-cause mortality occurred in 1,389 colchicine users (8.01%) compared to 1,920 non-users (11.07%), representing a 42% relative risk reduction (HR 0.58; 95% CI 0.53–0.62; P < 0.001, Fig 3A). Nonfatal stroke occurred in 744 colchicine users (4.29%) and 742 non-users (4.28%), with no significant difference between groups (HR 0.91; 95% CI 0.81–1.01; P = 0.076, Fig 3B). Nonfatal myocardial infarction occurred in 328 colchicine users (1.89%) and 256 non-users (1.48%), with no significant difference between groups (HR 1.15; 95% CI 0.96–1.37; P = 0.121, Fig 3C). Cause-specific mortality analysis revealed that the reduction in all-cause mortality was not driven by cardiovascular death (HR 0.95, 95% CI 0.78–1.16, P = 0.614), but rather by cancer-related death (HR 0.38, 95% CI 0.32–0.44, P < 0.001) and infection-related death (HR 0.56, 95% CI 0.43–0.72, P < 0.001) (Table B in S1 File).

**Table 2:** Cox analysis in matched cohort.

	Colchicine User (N = 17,337)	Non-user (N = 17,337)	HR (95% CI)	*P* value	aHR (95% CI)‡	*P* value^‡^
**Primary outcome^†^**	**2,170 (12.52)**	**2,606 (15.03)**	**0.71 (0.66–0.75)**	**<.0001**	**0.60(0.55–0.65)**	**<.0001**
**All cause death**	**1,389 (8.01)**	**1,920 (11.07)**	**0.58 (0.53–0.62)**	**<.0001**	**0.56(0.51–0.61)**	**<.0001**
**Nonfatal stroke**	**744 (4.29)**	**742 (4.28)**	**0.91 (0.81–1.01)**	**0.0762**	**0.75(0.64–0.89)**	**0.0007**
**Nonfatal MI**	**328 (1.89)**	**256 (1.48)**	**1.15 (0.96–1.37)**	**0.1211**	**0.85(0.65–1.13)**	**0.2729**

* Data are presented as n (%).

^†^ Composite outcome includes all-cause death, nonfatal stroke, and nonfatal MI.

^‡^ Adjusted hazard ratios from doubly robust analysis: Cox proportional hazards model additionally adjusted for age and sex within the PSM cohort.

* CI, confidence interval; HR, hazard ratio; MI, myocardial infarction.

**Fig 2:**
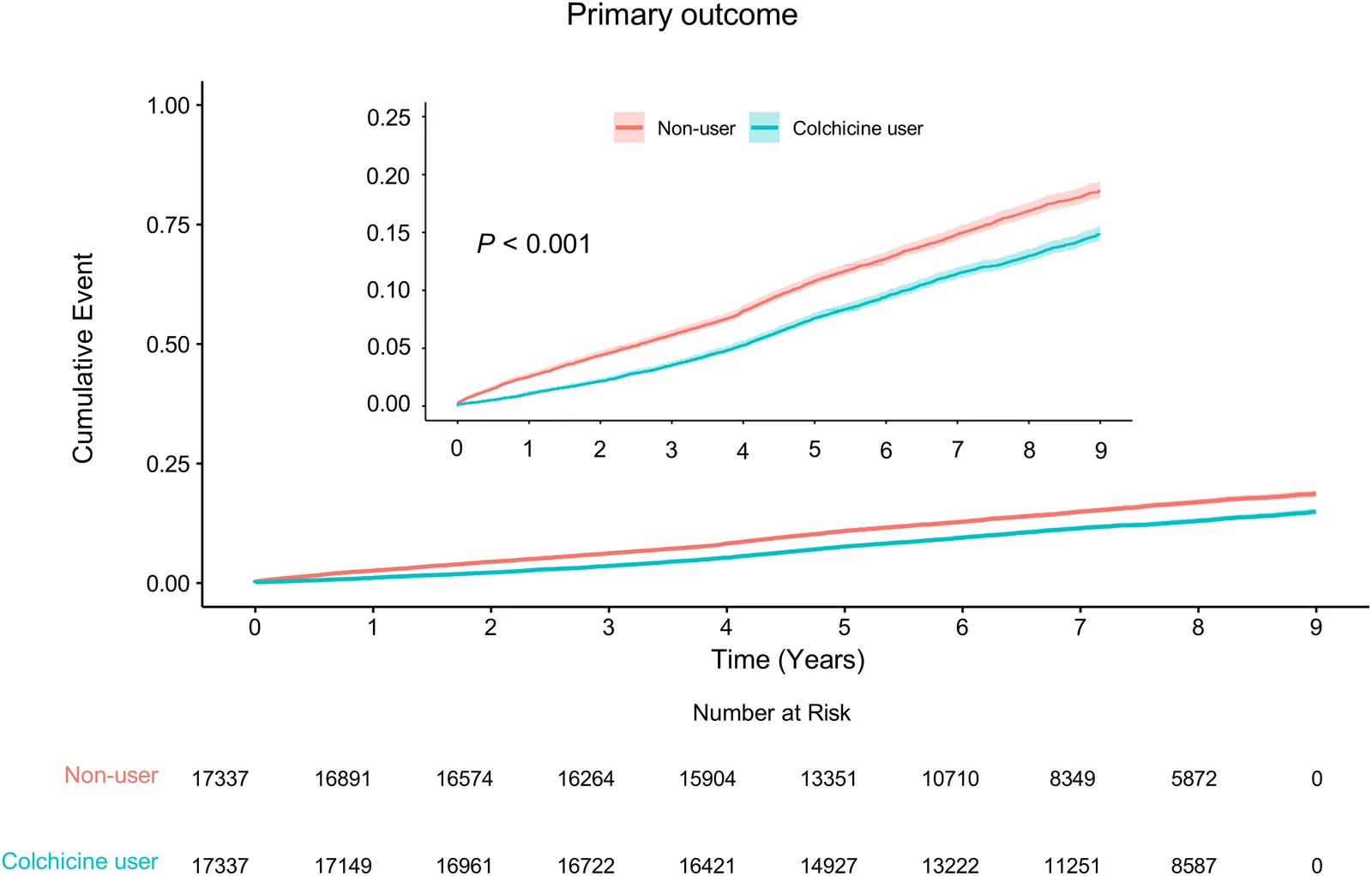
Kaplan-Meier curves for primary composite outcome.

**Fig 3:**
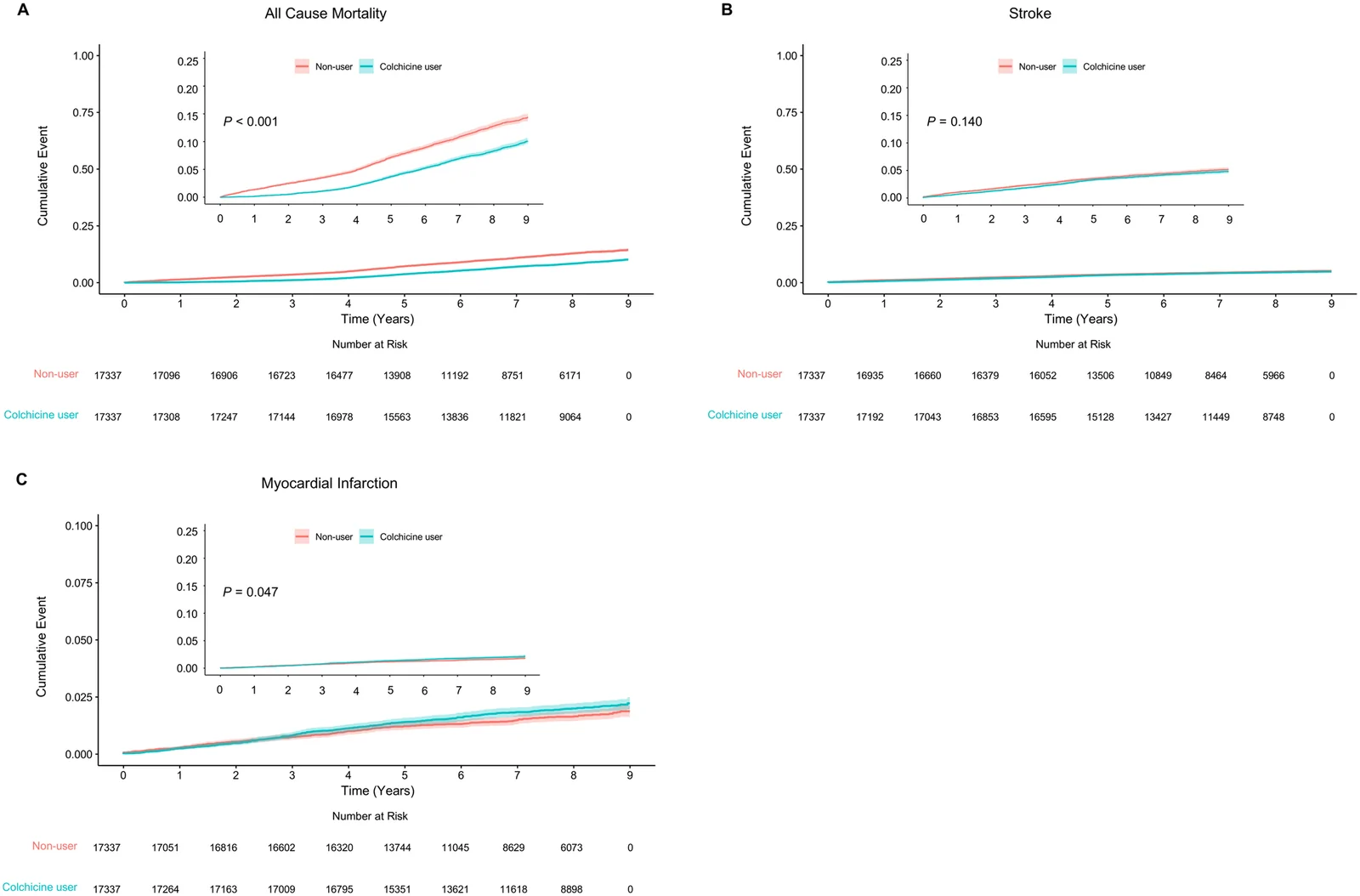
Kaplan-Meier curves for secondary outcomes.

### Sensitivity analyses

For the primary composite outcome (HR 0.71, 95% CI 0.66–0.75), the E-value was 2.17 (E-value for the lower confidence interval bound: 2.00). For all-cause mortality (HR 0.58, 95% CI 0.53–0.62), the E-value was 2.84 (E-value for the lower confidence interval bound: 2.61). In the doubly robust analysis, further adjustment for age and sex within the PSM cohort yielded an adjusted HR of 0.60 (95% CI 0.55–0.65, P < 0.001) for the primary composite MACE, 0.56 (95% CI 0.51–0.61, P < 0.001) for all-cause mortality, 0.75 (95% CI 0.64–0.89, P < 0.001) for stroke, and 0.79 (95% CI 0.69–0.90, P < 0.001) for composite MACE (cardiovascular death, stroke, MI) (Table 2).

In the landmark analysis at 365 days, colchicine use was associated with lower all-cause mortality (HR 0.67, 95% CI 0.62–0.73, P < 0.001) and with a lower rate of the primary composite outcome (HR 0.76, 95% CI 0.71–0.81, P < 0.001), and both associations were statistically significant. Stroke did not differ between the two groups and the difference was not statistically significant (HR 0.99, 95% CI 0.87–1.12, P = 0.823). Myocardial infarction also did not differ between the two groups and the difference was not statistically significant (HR 1.15, 95% CI 0.95–1.40, P = 0.144). The matched landmark cohorts contained 17,368 pairs for all-cause mortality, 17,202 pairs for stroke, 17,334 pairs for myocardial infarction, and 17,138 pairs for the primary composite outcome (Table C in S1 File).

## Discussion

In this nationwide, population-based cohort study of patients with both type 2 diabetes mellitus and gout without prior coronary artery disease, colchicine use was associated with a significant 29% reduction in the composite MACE. This benefit was primarily driven by a significant 42% reduction in all-cause mortality, while individual atherosclerotic endpoints (myocardial infarction and stroke) did not reach statistical significance in the primary matched analysis. In the doubly robust analysis, stroke reached statistical significance (adjusted HR 0.75, 95% CI 0.64–0.89, P < 0.001). However, this result was not reproduced in the landmark analysis, in which stroke did not differ between the two groups (HR 0.99, 95% CI 0.87–1.12, P = 0.823). Because the stroke findings were not consistent across analytic approaches, we regard them as hypothesis-generating rather than as evidence of a treatment effect. These findings were consistent in both the propensity score-matched cohort and the entire unmatched population. Cause-specific mortality analysis revealed that the mortality reduction was primarily attributable to cancer-related and infection-related deaths rather than cardiovascular death, suggesting that the benefit of colchicine in this primary prevention population may operate through different pathways than those observed in secondary prevention trials [5,7]. While prior RCTs in secondary prevention populations were designed to detect atherosclerotic event reduction over relatively short follow-up periods, our study with longer follow-up in a primary prevention population with high baseline inflammatory burden (diabetes plus gout) may have been better positioned to capture mortality benefits operating through non-atherosclerotic inflammatory pathways. Rather than contradicting RCT findings, our results may complement them by revealing additional dimensions of colchicine's anti-inflammatory effects.

Our findings suggest the potential to extend cardiovascular benefits of colchicine from the secondary prevention to primary prevention. Previous randomized controlled trials demonstrated that colchicine reduces cardiovascular events in patients with established ASCVD following myocardial infarction or with chronic coronary syndromes [5–7]. However, evidence for primary prevention in patients without prior ASCVD remains limited [9]. To study primary prevention, we excluded patients with prior coronary artery disease. We focused on diabetic patients because they have high cardiovascular risk and are most likely to benefit from primary prevention strategies. We included only gout patients because colchicine is primarily prescribed for gout. Both the colchicine users and non-users had gout, creating comparable groups and minimizing selection bias. The absence of significant reduction in individual cardiovascular endpoints in our study likely reflects the lower atherosclerotic event rates inherent to a primary prevention cohort without prior coronary artery disease, which limits statistical power for these endpoints. The cause-specific mortality analysis provides important insight into the nature of the observed mortality benefit. The significant reductions in cancer-related and infection-related deaths, without a corresponding reduction in cardiovascular death, suggest that colchicine's broad anti-inflammatory properties may confer survival benefits beyond atherosclerosis in patients with chronic inflammatory conditions such as diabetes and gout. These findings provide a mechanistic explanation for the mortality benefit. The CANTOS trial demonstrated that IL-1β inhibition was associated with reduced cancer mortality, particularly lung cancer [24,25]. Since colchicine modulates the NLRP3 inflammasome pathway, which is upstream of IL-1β, this provides a biological rationale for the cancer-related mortality reduction observed in our study [26–28]. However, these cause-specific findings should be considered exploratory, and the possibility of residual confounding, including healthy user bias, cannot be entirely excluded. Future prospective studies are needed to clarify the mechanisms underlying these observations.

Because colchicine users were identified from prescriptions that occurred after the gout diagnosis while follow-up began at the gout diagnosis for both groups, the primary analysis allowed immortal time. We therefore performed a landmark analysis at 365 days. The association with all-cause mortality was attenuated from a hazard ratio of 0.58 to 0.67, and the association with the composite outcome was attenuated from 0.71 to 0.76, yet both remained statistically significant. The median interval between the gout diagnosis and the first colchicine prescription was 0 days, because colchicine treats an acute gout flare at the time of diagnosis in Korean practice. Therefore, immortal time was absent for most colchicine users, and this is consistent with the attenuation that we observed.

Myocardial infarction occurred more often in colchicine users in absolute numbers, but the difference was not statistically significant in the primary analysis (HR 1.15, 95% CI 0.96–1.37, P = 0.121) or in the landmark analysis (HR 1.15, 95% CI 0.95–1.40, P = 0.144). In the landmark cohort the cumulative incidence of myocardial infarction was 1.7% in colchicine users and 1.2% in non-users, a ratio of about 1.4, whereas the hazard ratio was 1.15. Death was censored in this analysis, and colchicine users had lower mortality. Therefore, colchicine users remained at risk for a longer period and had more opportunity to experience a myocardial infarction. A competing risk framework would be needed to separate these two effects.

The main strengths of our study include the nationwide design making our findings applicable to real-world practice, large sample size giving us enough power to find important differences, complete recording of all medical visits through required national insurance system, careful selection criteria reducing bias, and propensity score matching to reduce confounding. However, this study has a few limitations. First, as a retrospective observational study using an administrative claims database, we cannot establish a causal relationship. Although our PSM model included a comprehensive set of covariates including demographics, comorbidities, and concomitant medications, clinical laboratory values such as HbA1c and serum uric acid levels were not available in the NHIS claims data. Diabetes duration could not be precisely ascertained, as the database records the date of first diagnostic code rather than the true clinical onset. However, the inclusion of diabetes medication classes (metformin, sulfonylureas, insulin) in the propensity score model served as surrogate markers for disease severity and glycemic control intensity. Furthermore, E-value analysis demonstrated that an unmeasured confounder would need to be associated with both the exposure and outcome by a risk ratio of at least 2.17-fold (for MACE) or 2.84-fold (for all-cause mortality) to fully explain away the observed associations, which suggests that unmeasured confounding alone is unlikely to explain the observed associations. A doubly robust analysis adjusting for residual imbalance in age and sex also yielded consistent results, further supporting the validity of our findings. Second, colchicine was prescribed to treat gout, not to prevent cardiovascular disease. In Korea, colchicine is reimbursed by the National Health Insurance only for gout-related indications, and prescribing it for other purposes is subject to claims denial and financial penalties for physicians. Therefore, colchicine use in Korean clinical practice is inherently linked to gout diagnosis, and our study was designed to reflect this real-world prescribing context. Since colchicine is not prescribed to non-gout patients in Korea, a comparison group of diabetic patients without gout receiving colchicine does not exist in routine practice. This is not a limitation of our dataset, but rather a reflection of the actual prescribing environment. Our findings may not apply to patients without gout, and future randomized controlled trials are needed to determine whether the cardiovascular benefits of colchicine observed in secondary prevention trials extend to primary prevention in diabetic patients without gout. Third, the benefit in the primary outcome was mainly due to reduced all-cause mortality, with no significant differences in myocardial infarction or stroke in the matched cohort. However, this likely reflects the lower atherosclerotic event rates inherent to a primary prevention cohort, which limits statistical power for individual cardiovascular endpoints. The doubly robust analysis demonstrated a statistically significant reduction in stroke (adjusted HR 0.75, 95% CI 0.64–0.89, P < 0.001), but the landmark analysis did not (HR 0.99, 95% CI 0.87–1.12, P = 0.823). We therefore do not claim a stroke benefit. Furthermore, the cause-specific mortality analysis provided mechanistic insight, revealing that the mortality benefit was primarily driven by cancer-related and infection-related deaths rather than cardiovascular death, which may represent a unique contribution of this study in a population with high baseline inflammatory burden. Fourth, our exposure definition classified any patient with at least one colchicine prescription as a user, which represents a broad definition encompassing highly variable treatment durations. The mean prescription duration of 192.12 days (standard deviation, 406.01 days) suggests substantial heterogeneity in colchicine exposure. This conservative definition may have biased results toward the null, and the fact that significant associations were still observed suggests that the true treatment effect could be even larger with sustained exposure. Future studies with dose-response analyses or minimum exposure thresholds would help clarify the optimal treatment duration for cardiovascular protection. While propensity score matching reduced these differences, some residual confounding by disease severity may remain. Fifth, we excluded patients with prior coronary artery disease but not patients with prior stroke, other cerebrovascular disease, or peripheral artery disease. The cohort therefore represents primary prevention with respect to coronary artery disease rather than to all atherosclerotic cardiovascular disease. Randomized controlled trials designed to confirm colchicine for primary prevention are needed.

## Conclusions

In patients with diabetes and gout without prior coronary artery disease, colchicine use was associated with lower all-cause mortality, and this association persisted in a landmark analysis that addressed immortal time bias. The reduction in the composite outcome was driven by mortality rather than by myocardial infarction or stroke, and the mortality reduction was attributable to non-cardiovascular causes. Therefore, these findings do not establish a cardiovascular benefit of colchicine in primary prevention. The observational design precludes causal inference, and randomized controlled trials are warranted.

## Supporting information

S1 File Supplementary tables. Table A. Colchicine prescription duration. Table B. Cox analysis in the matched cohort. Table C. Cox analysis in the landmark cohort at 365 days.
(DOCX)
