# Supplementary material for: Colchicine use was associated with reduced all-cause mortality in patients with diabetic gout without prior coronary artery disease: A propensity-matched nationwide cohort study
Source: PLoS One. 2026 Nov 11;21(11):e0362218. doi: 10.1371/journal.pone.0362218 (PMC13754227; doi:10.1371/journal.pone.0362218)
Supplement: Supplementary file 1 [file pone.0362218.s001.docx]

**Table A. Colchicine Prescription Duration**

| **Variable** | **N** | **Mean** | **SD** |
| --- | --- | --- | --- |
| **Total prescription (days)** | 17,337 | 192.12 | 406.01 |

* Values represent cumulative colchicine prescription days during the entire follow-up period among propensity score-matched colchicine users.

* SD, standard deviation.

**Table B. Cox analysis in matched cohort**

|  | **Non-user**  **(N=17,337)** | | **Colchicine user**  **(N=17,337)** | | **HR (95% CI)** | **P value** |
| --- | --- | --- | --- | --- | --- | --- |
| **All cause death** | 1920 | 11.07% | 1389 | 8.01% | 0.58(0.53-0.62) | <0.001 |
| **CV death** | 233 | 1.34% | 252 | 1.45% | 0.95(0.78-1.16) | 0.614 |
| **Cancer death** | 684 | 3.95% | 318 | 1.83% | 0.38(0.32-0.44) | <0.001 |
| **Infection-related death** | 169 | 0.97% | 110 | 0.63% | 0.56(0.43-0.72) | <0.001 |
| **Metabolic/endocrine death** | 51 | 0.29% | 43 | 0.25% | 0.67(0.43-1.05) | 0.079 |
| **Else** | 456 | 2.63% | 358 | 2.06% | 0.66(0.56-0.77) | <0.001 |

* Reference: Non-user group. HR, hazard ratio; CI, confidence interval; CV, cardiovascular.

**Table C. Cox analysis in landmark cohort at 365 days**

|  | **N per group** | **Non-user** | **Colchicine user** | **HR (95% CI)** | **P value** |
| --- | --- | --- | --- | --- | --- |
| **All cause death** | 17,368 | 1,725 (9.9) | 1,421 (8.2) | 0.67 (0.62–0.73) | <0.001 |
| **Stroke** | 17,202 | 573 (3.3) | 604 (3.5) | 0.99 (0.87–1.12) | 0.823 |
| **Myocardial infarction** | 17,334 | 211 (1.2) | 289 (1.7) | 1.15 (0.95–1.40) | 0.144 |
| **Composite MACE** | 17,138 | 2,213 (12.9) | 1,948 (11.4) | 0.76 (0.71–0.81) | <0.001 |

* Reference: Non-user group. The landmark time was 365 days after the gout diagnosis. Patients who died or developed the outcome of interest before the landmark were excluded, and propensity score matching was repeated within each landmark cohort, so the number of patients differs by outcome. Deaths after the landmark were censored in the analyses of stroke and myocardial infarction. Data presented as n (%). HR, hazard ratio; CI, confidence interval; MACE, major adverse cardiovascular events.
